# LC-MS metabolomics from study design to data-analysis – using a versatile pathogen as a test case

**DOI:** 10.5936/csbj.201301002

**Published:** 2013-02-06

**Authors:** Maya Berg, Manu Vanaerschot, Andris Jankevics, Bart Cuypers, Rainer Breitling, Jean-Claude Dujardin

**Affiliations:** a Unit of Molecular Parasitology, Department of Biomedical Sciences, Institute of Tropical Medicine, Nationalestraat 155, 2000 Antwerp, Belgium; b Institute of Molecular, Cell and Systems Biology, College of Medical, Veterinary and Life Sciences, University of Glasgow, Joseph Black Building B3.10, G11 8QQ Glasgow, UK; c Groningen Bioinformatics Centre, Groningen Biomolecular Sciences and Biotechnology Institute, University of Groningen, Nijenborgh 7, 9747 AG Groningen, The Netherlands; d Faculty of Life Sciences, Manchester Institute of Biotechnology, University of Manchester, 131 Princess Street, Manchester M1 7DN, UK; e Department of Biomedical Sciences, University of Antwerp, Universiteitsplein 1, 2610 Antwerp, Belgium

**Keywords:** mass spectrometry, HILIC, *Leishmania*, unicellular trypanosomatid parasites, global molecular profiles, systems biology

## Abstract

Thanks to significant improvements in LC-MS technology, metabolomics is increasingly used as a tool to discriminate the responses of organisms to various stimuli or drugs. In this minireview we discuss all aspects of the LC-MS metabolomics pipeline, using a complex and versatile model organism, Leishmania donovani, as an illustrative example. The benefits of a hyphenated mass spectrometry platform and a detailed overview of the entire experimental pipeline from sampling, sample storage and sample list set-up to LC-MS measurements and the generation of meaningful results with state-of-the-art data-analysis software will be thoroughly discussed. Finally, we also highlight important pitfalls in the processing of LC-MS data and comment on the benefits of implementing metabolomics in a systems biology approach.

## 1. Introduction

During the past few years, technologies for global molecular profiling have revolutionised our understanding of biology with improved sequencing technologies leading to major advances in genomic and transcriptomic studies [1] and more selective and sensitive mass spectrometers driving a rapid expansion of proteomic and metabolomic studies [2, 3]. Within parasitology, this resulted, for instance, in the relatively rapid whole genome sequencing of a whole range of pathogens (*Plasmodium*, *Leishmania*, *Trypanosoma, Schistosoma*, …) [4–9] and the application of metabolic fingerprints for the identification of biomarkers in parasitic infection [10, 11]. While genomics and transcriptomics study the starting point of the molecular cascade leading towards a specific phenotype, metabolomics can study the ultimate expression of the genotype and is thus the profiling technology that works closest to the eventual phenotype [12]. In addition, genome and proteome studies often struggle with the functional annotation of identified sequences [13], while the metabolome consists of relatively few low molecular weight molecules – called metabolites –, many of which are key actors of cellular processes which are universal across organisms, such as energy metabolism (e.g., glycolysis and the tricarboxylic acid (TCA) cycle) or the catabolism and anabolism of universal cellular components (e.g., amino acid biosynthesis and the urea cycle). Metabolomics is of particular interest for the study of our favourite model organisms, the unicellular trypanosomatid parasites, including pathogens such as *Trypanosoma* and *Leishmania*, as their gene expression is regulated almost exclusively at the post-transcriptional level [14, 15], so that genome and transcriptome studies might have limitations, in particular when studying the rapid effects of drug treatment or the mechanisms of drug resistance.

*Leishmania* is a protozoan parasite that alternates between two major developmental stages: flagellated promastigotes that occur in the sandfly vector and non-motile amastigotes that develop within the phagolysosome of mammalian macrophages [16]. This parasite has proven to be exceptional in many ways: (i) its chromosomes can vary in copy number between strains, and important genes can be amplified as circular extrachromosomal episomes [5, 17]; (ii) it has a unique thiol redox metabolism, lacking glutathione reductase, but possessing trypanothione and trypanothione reductase [18, 19]; and (iii) antimony drug resistance of the parasite has been associated with an increased fitness of the parasite instead of the usual fitness cost [20]. After unravelling its genome [5, 9, 21] and gaining a better understanding of its transcriptome [22] and proteome [23], the *Leishmania* metabolome is now the focus of several research projects. For example, recent studies uncovered metabolic changes that occur throughout *in vitro* promastigote growth [24], but also between natural drug sensitive and drug resistant *Leishmania* strains [25]. In this minireview we will discuss the technique of LC-MS metabolomics from sampling to generating meaningful results, highlighting important pitfalls and discussing the benefits of a systems biology approach, using *Leishmania* as an illustrative example of a complex model organism.

## 2. The LC-MS platform

The favorite technology for global metabolic profiling (metabolomics) are so-called hyphenated MS platforms, such as gas chromatography-mass spectrometry (GC-MS), liquid chromatography-mass spectrometry (LC-MS) or capillary electrophoresis-mass spectrometry (CE-MS) [26]. Alternatively, NMR spectroscopy, direct infusion atmospheric pressure ionization (API) MS, and other methods, such as Raman spectroscopy and Fourier transform infra-red spectroscopy, can be used for higher throughput but less specific metabolomics screening experiments (fingerprinting) (for a comparison see [13]). The selection of the platform is always a compromise between sensitivity, speed and chemical selectivity and coverage of the relevant subset of the metabolome [27]. One must bear in mind that the chemical diversity and the range of concentration of different metabolites is very diverse, therefore no single platform provides a complete coverage of the metabolome [28].

For *Leishmania*, only LC-MS and GC-MS metabolomics studies have been reported [24, 25, 29–31]. Chromatographic separation by LC or GC has two main advantages when compared to direct-infusion MS: (i) it separates isomers (metabolites of a same mass) which would appear as indistinguishable entities in downstream MS analysis; and (ii) it minimizes ion suppression in which a more easily ionizable species masks the presence of a less ionizable one [32] hence allowing a higher quantitative accuracy [33]. The hyphenation of MS, i.e. its combination with a chromatographic separation, greatly increases the quality of the raw data generated and the number of metabolites to be detected, but it also increases the analysis time [27]. A detailed comparison between GC-MS and LC-MS – the two main separation methods in metabolomics – is described elsewhere [26, 28]. In short, compared to LC-MS, GC-MS analysis involves a more complex sample preparation, since it is only capable of analyzing volatile compounds or those that can be made volatile by derivatization [34]. In addition, many polar compounds are not detectable by GC-MS, and due to the electron ionization (EI) technique used in GC-MS, only the most abundant positively charged ions are measured [26, 28]. However, GC-MS generates reproducible fragmentation patterns, for which fragment databases exist (that can be shared between investigators), and produces stable retention times, which can be matched with existing libraries containing retention time information, for a huge array of analytes [13]. This makes it much easier to verify the identification of detected metabolites in GC-MS. The LC-MS situation is more complex: the atmospheric pressure ionization techniques (APCI, ESI) produce both positively and negatively charged ions, but suffer more from matrix effects and ionization suppression or enhancement. In addition, while LC-MS also generates characteristic retention times for each metabolite, which assists in metabolite identification, these retention times are more difficult to reproduce and compare between laboratories and library matching is still at an early stage. In reality, choosing between a GC-MS and an LC-MS platform is most often determined by the availability of a platform and existing collaborations.

## 3. From sample preparation to LC-MS measurement

### 3.1. Choice of life stage

Many components of the metabolome change only very slowly throughout life, making selected metabolites popular biomarkers in human medicine (cholesterol being a very common example). However, at the cellular level changes can be much more rapid, and between different cell types or developmental stages, large fractions of the metabolome can be drastically rearranged. This has important consequences for the choice of sample for a metabolomics analysis. Many unicellular pathogens, especially those that are transmitted through a vector, have different life forms. Choosing the correct life stage of the organism under study greatly depends on the research question. Are the suspected differences expected to be present throughout all life stages or only at one specific stage? In the case of *Leishmania* for example, the intracellular amastigotes are the most clinically relevant form to study, as only this form occurs in the human host. However, amastigotes have as yet not been thoroughly studied at the metabolomics level due to several technical constraints (difficulty to separate its metabolome from that of the host cell, quick transformation to promastigote life stage upon isolation, difficulty of obtaining sufficient quantities) [35]. Free-living pathogens, such as trypanosomes belonging to the subgenus *Salivaria*, create fewer problems concerning the choice of life stage for metabolomics studies, since both the procyclic (fly vector) and the bloodstream form (human host) can be easily extracted [36, 37]. The extracellular promastigote form of *Leishmania*, which naturally occurs in the vector, is easier to culture *in vitro* and is therefore also the most studied life form of the parasite in metabolomics and other studies.

Another issue often affecting metabolomics studies is that cells come in different sizes: when comparing the metabolic profile of two samples with a significant difference in cell size, the eventual results can be skewed, with the larger cell showing generally increased metabolite levels which is superimposed upon the metabolite changes of interest. In contrast to, for example, transcriptomics, no commonly accepted standard procedure is available for correcting this bias. Normalizing the metabolomics results according to the cell size might be recommendable if such differences are known to occur. Although such a normalization method may seem justified to biologists, many LC-MS specialists feel that this is perilous because LC-MS signals do not always scale linearly (see further in section 5). The semi-quantitative nature of LC-MS measurements allows only comparison of the same (!) metabolites between different samples within the same measurement block, and not comparison of the quantity of different metabolites within a given sample. Hence, one single normalization factor for all metabolites could over-or under-correct the intensities of metabolites with different physicochemical properties. Nevertheless, protein content normalization has already been applied when comparing the metabolic profile of one *Leishmania* strain at different stages in the promastigote growth curve [24]. In this study, it was shown that transformation to the metacyclic form (the smaller infective form) was accompanied by a decrease in protein content, which is thought to correlate with the decrease in cell size. Hence, by determination of the total protein content present in a sample with a commercially available kit, differences in cell size can be corrected.

### 3.2. Sampling

A sampling protocol that minimizes the biological and technical variability is indispensable for any biological metabolite profiling study. This is also the case for metabolomics, since the metabolome can change very rapidly, for example in response to differentiation processes or subtle changes in the environment (such as temperature fluctuation, osmotic stress, or nutrient depletion). Thorough preparation of the whole sampling pipeline will be imperative to ensure a swift sample preparation that minimizes the induction of additional biological or technical variability induced by the sampling procedure itself. To reduce the technical variability throughout the sampling procedure, it is of utmost importance to bring the metabolism of the cells rapidly to a halt and avoid leaking of metabolites during the various washing steps before the actual metabolite extraction. Rapid quenching of the metabolism of *Leishmania*, or related unicellular protozoan parasites, without freezing the culture can be best achieved by immersing the culture flask in an ethanol bath cooled with dry ice at 0-4 °C for no more than 60 s [24, 25, 30, 31, 33, 36, 38, 39]. All further manipulations should be done on ice or at ice-cold temperatures (0-4°C) in order to maintain this quenched status. After washing the required number of cells, typically between 4E + 07 to 1E + 08, by repeated centrifugation and resuspension in cold PBS – 3 washing steps have proven optimal with minimal metabolite leakage [39] –, the cells are to be disrupted and the metabolites extracted. Several extraction methods specifically for *Leishmania* samples have been thoroughly compared by t'Kindt *et al*., showing that the addition of 200 µl 1:3:1 chloroform/methanol/water [30] and incubation in a Thermomixer for 1 hr (14000 rpm, 0°C) is superior to other heating, mixing, vortexing or milling methods [39]. The metabolites can then be extracted from the cell debris by centrifugation and stored at –80°C. Internal standards (stable isotopes) can be spiked into all samples to detect variability in sample processing (spiking should be done before extraction) and/or technical reproducibility issues of the analysis platform (spiking should be done as a last step before storage). This standard protocol is now commonly used for both *Leishmania* and *Trypanosoma* metabolomics studies [24, 25, 33, 36, 38, 39]. If lipids are of special interest, different organic solvents for optimal extraction of lipids such as 2:1 chloroform/methanol should be used and might require other disruption methods such as sonication [40].

In addition to various control samples, and depending on the study outline, other samples can be obtained simultaneously as well. If a parallel genomic or proteomic study is planned, for example, it is best to prepare these samples at the same time as the metabolomics study, due to the variability of the metabolome and the plasticity of the *Leishmania* genome [5, 17]. This significantly facilitates later integration of the metabolome and the genome, transcriptome and/or proteome results into a general systems biology interpretation. Furthermore, when processing several strains together, the genome sequence can also be used as a quality control to confirm the identity of the material used.

### 3.3. Sample storage

Just before storage of the metabolomics samples at –80°C, samples should be deoxygenated with a gentle stream of nitrogen gas for 1 min prior to tube/vial closure [39]. The effect of storing serum and urine samples at 4°C for 0 h or 24 h prior to storage at –80°C has been shown to be small: the observed variance between samples due to storage at 4°C for 24 h was of the same magnitude as the analytical variance associated with replicate analysis per sample [41]. Dunn *et al*. recommend analyzing samples within 2 years of sample collection and avoiding multiple freeze-thawing cycles of a single aliquot [34]. Optimally, a sample should be opened only once and, if needed, multiple aliquots of the same subject can be collected [34]. To our knowledge, reports on the stability of metabolites present in parasite samples, or plasma, serum, urine or cerebrospinal fluid samples for that matter are rather scarce [34, 41–43].

### 3.4. Sample list set up

The decision which samples to measure by LC-MS and in which order is far from trivial, as it affects the accurate assessment of biological and technical variability.

**Figure 1:**
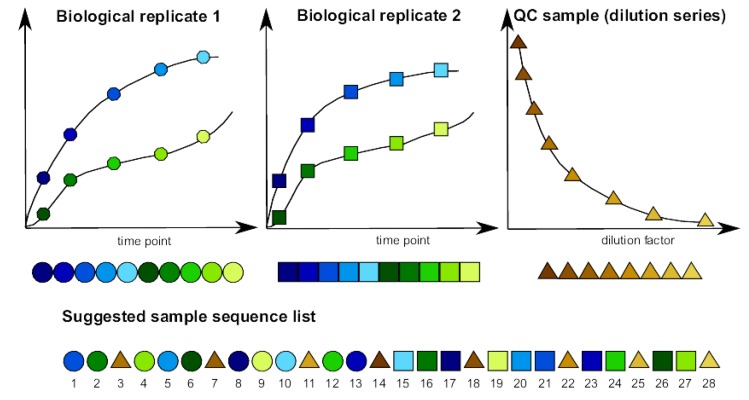
Recommended sample sequence for samples from two different experimental conditions (condition 1 = shades of blue, condition 2 = shades of green) measured at five different time points. Each condition has two biological replicates. The y-axis represents the measured intensities of the biological replicate, whereas the x-axis represents the five different time points. Samples from a dilution series of a quality control pooled reference samples (shades of brown) are interspersed at regular intervals in the suggested sample sequence.

Most metabolomics studies include three or four biological replicates of each experimental treatment [24, 25, 30, 44, 45]. Beside the biological replicates, a series of other samples should be included in each LC-MS run. First of all, a reference sample should be injected at least four to eight times to equilibrate the analytical platform and assess the reproducibility of subsequent runs [46]. Preferably, the reference sample is similar to the actual samples of interest in complexity and composition. In addition, commercially available authentic standards can be added to the measurement series to compare retention time for metabolite identification. For example, Creek *et al*. describe the use of 2 mixtures of authentic standards (127 metabolites in total), which can be used to predict retention times also for compounds that are not included in the mixture [44]. Finally, a dilution series of a pooled sample of all extracts can be included, which will help to filter out a substantial part of spurious signals [47]. The measurements of both the standards and the reference samples should be regularly distributed throughout the sample list so they can be used for quality control to assess LC-MS stability [34, 48]. Additional controls can include cell-free growth medium and extraction solvent blanks to filter out contaminant peaks by ‘blank’ subtraction. The order of all these samples should be well considered: randomization of the different samples within blocks of four biological replicates alternated with quality control samples is recommended; this will allow detecting systematic variability throughout the LC-MS measurement [25]. Figure 1 illustrates a recommended sample sequence, based on a dilution series of quality control samples and randomization of analytical samples within blocks (shown for two biological replicates per sample). For example, if all biological replicates of condition 1 are measured first, followed by all biological replicates of condition 2, technical issues during the LC-MS experiment (in particular the unavoidable column degradation) would result in a confounding of experimental and temporal factors, seriously interfering with the later statistical interpretation of the data. By randomizing the biological replicates in a well-considered way, this can be largely avoided (Figure 1). The quality control samples which alternate with the biological replicates are used to detect and potentially correct for these technical issues.

### 3.5. LC-MS measurements

#### Chromatographic separation

**Figure 2:**
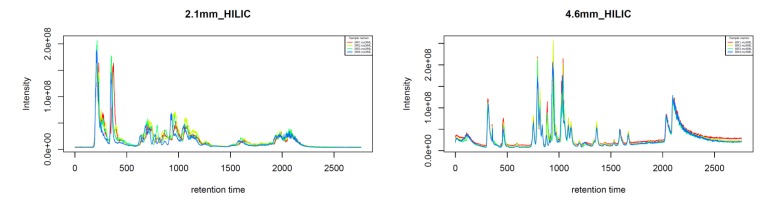
Total Ion Chromatograms of four biological replicates of the same *Leishmania* sample, measured on both the 2.1 mm HILIC column (left) and the 4.6 mm HILIC column (right) coupled to an Orbitrap Exactive mass spectrometer with analytical conditions as described in [25]. The chromatogram was plotted in IDEOM (“TIC checker” functionality) and shows that the 4.6 mm HILIC column provides considerably more reproducible retention times, facilitating downstream analysis and metabolite identification.

The most widely used liquid chromatography system in metabolomic research on *Leishmania* and related protozoan parasites [24, 25, 37, 39] is the HILIC column (hydrophilic interaction liquid chromatography): it allows polar metabolites to be retained, whereas lipophilic metabolites elute relatively rapidly from the column. This is a significant advantage compared to reversed-phase columns, from which lipids are difficult to elute and can accumulate and cause ion suppression by their background bleed [37]. The performance of two HILIC columns with a different inner diameter (2.1 mm versus 4.6 mm) has been compared and showed that the number of putatively identified metabolites dropped nearly two-fold for the wider column (from 390 to 220). Using the 2.1 mm HILIC column, 20% of the predicted metabolome of *Leishmania* could be detected [39]. However, it was also notable that the narrower column behaved in a less reproducible way, especially in terms of retention time drift (Figure 2).

For large batch analysis, the 4.6 mm HILIC might thus be the preferable column. To obtain a more complete coverage of the metabolome, measurements on different types of columns can be combined. For example, since lipids are considered to be biologically important in *Leishmania* drug metabolism [25], lipidomics studies in *Leishmania* are becoming of increasing importance to unravel the mechanisms of drug resistance [40]. These studies use different types of columns, ranging from HILIC [33] to normal phase [40].

#### Mass spectrometry

For an overview of the existing ion separation methods we refer to Watson [49]. In summary, the Orbitrap mass spectrometer is the most sensitive instrument currently applied in general metabolomics studies: it combines ultra-high mass accuracy (<1ppm) and resolution (>100,000) with a high dynamic range (approx. 10^5^), allowing unambiguous assignment of a molecular formula to many observed masses [28]. LC-MS generally analyzes samples in both positive (ESI + ) and negative ion mode (ESI–), as they provide complementary data. The Orbitrap Exactive configuration is well-suited for this kind of analysis, since it has a positive–negative polarity switch mode which reduces analysis time, amount of sample needed and issues related to combining the two modes afterwards if they were not recorded simultaneously (such as retention time drift, see section 5). Time-of-Flight instruments (TOF) are also compatible with chromatographic systems interfaced to an ESI source, but linear dynamic ranges are around 10^3^ and the resolving power is limited to 3 ppm [49].

## 4. Data-analysis

Despite recent advances in methodology, metabolomics still presents a number of challenges, including both technological issues and limitations of data interpretation [50]. As typically a large amount of signals is detected, data complexity usually is so high that it is not possible to interpret data manually; hence, specific software tools and algorithms are needed. These types of analysis also require fast processors and huge storage capacity, typically in the terabyte range for large datasets. Comprehensive overviews of many of the existing tools for data processing in metabolomics have been presented recently [61–64].

**Figure 3:**
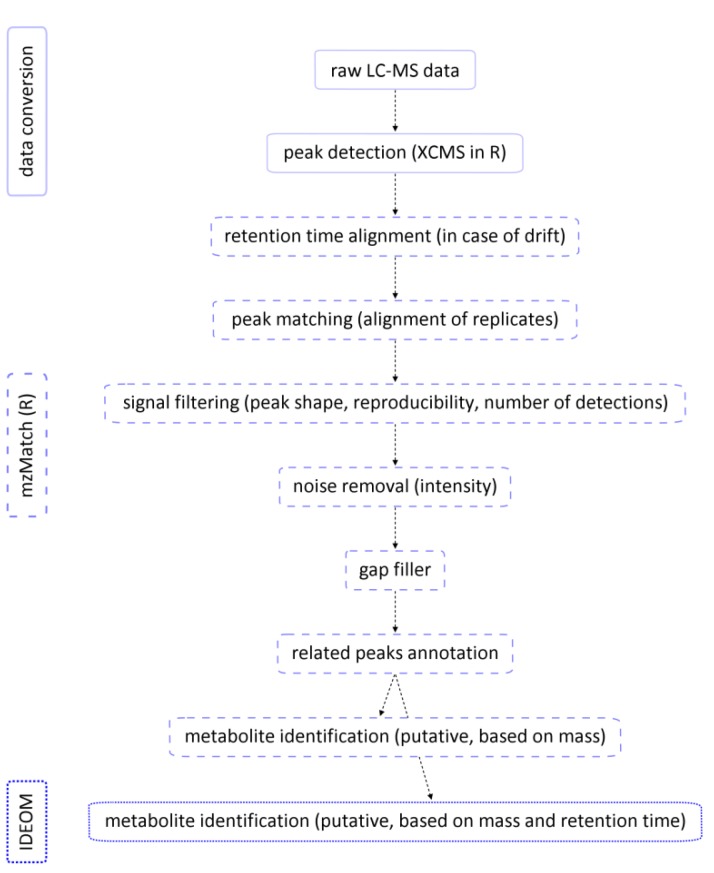
Schematic overview of an LC-MS metabolomics data processing pipeline.

General data processing steps include feature or peak detection [51], peak matching and several additional steps of signal filtering and noise removal [52] (Figure 3). For example, the peak matching step involves aligning of the chromatographic features between technical or biological replicates of a single sample. Peaks that are not detected in all technical replicates can be discarded from further analysis. Derivative signals such as isotopes, adducts, dimers and fragments, can be automatically annotated by correlation analysis on both signal shape and intensity patterns using software tools like CAMERA [53], PUTMEDID-LCMS [54] and mzMatch [55]. Such peaks are not discarded, but only flagged, so that their assigned annotations can be taken into account in the metabolite identification step.

Metabolite identification in LC-MS is mainly based on matching the detected mass with available mass databases and derivative signal annotations. In contrast to proteomics, efficient algorithms that can reasonably successfully predict and compare the mass fragmentation patterns for tandem MS spectra of metabolites (e.g. MetFrag [56]) still need further development. In addition, Creek *et al*. suggest applying a quantitative structure retention relationship (QSRR) model on authentic standard compounds to predict retention times of chemically similar compounds [44]. Including the predicted retention times in the identification step significantly improved metabolite identification by removing 40% of falsely identified compounds, which had the correct mass but inconsistent retention time.

As the nature of metabolic experiments and experimental design varies widely, there is a demand for software tools that could easily be adapted for the evolving demands of data processing, for example, adding extra data filtering tools or changing the order of a typical processing pipeline. A recent study by Jankevics *et al*. [45] illustrates the configurable software toolkit mzMatch for the complete processing of raw mass spectra, including steps for noise filtering and compound identification by matching mass databases [55]. The PeakML file format [55] used by mzMatch allows users to share data with other commonly used software packages such as XCMS [57], mzMine [58] and IDEOM [59], giving flexible access to an extended set of data processing tools. For instance, IDEOM is a user-friendly Excel interface to mzMatch which allows researchers to run a comprehensive pipeline for data-analysis and visualization from a graphical user interface within Microsoft Excel [59]. Efforts to develop such tool chains and (semi-) automated tools for data processing in unified data exploration platform will be a priority in the near future.

## 5. Current issues with LC-MS

A major drawback of LC-MS is that it only allows for semi-quantitative analysis. For example, LC-MS signals do not always scale linearly with metabolite concentrations, as has clearly been shown by dilution series [47]. The deviation from linearity strongly depends on (i) the type of column (HILIC versus C18; HILIC is more prone to variations in signal intensity); (ii) the concentration of the metabolite (ion suppression occurs more frequently with higher concentrations); and (iii) the loading capacities of the column. Therefore, it is important to always interpret the metabolite profiles in terms of relative quantification, where the raw peak height of a metabolite of interest is compared to the raw peak height of the same (!) metabolite in a reference sample or, for example, other samples in a time series. During the last few years, however, absolute quantification of selected compounds using ^13^C-labelled standards is gaining ground in global metabolomics studies [13, 28, 60], providing unique insights into the dynamics of metabolic fluxes, beyond the steady-state information gathered by routine mass spectrometry [60]. The Maven [61] and mzMatch-ISO packages can be used to process isotope-labeled data sets [http://mzmatch.sourceforge.net/untargeted_labelling.php].

The combination of LC-MS based metabolomics data that were collected over a longer period of time on the same platform and in the same laboratory remains problematic due to the systematic variability between LC-MS measurements [62]. This systematic variability includes variable ionization (influenced by many factors, such as co-elution of other metabolites, salts, pH of the mobile phase etc.), drift in retention time (column degradation or replacement), and drift in mass calibration (changes in temperature and electronic circuitry). Samples that need to be compared should therefore preferably be measured in the same run (and if possible in randomized order), although total run length should be limited, because contamination will cause drifts in the measured response and retention time over relatively short analysis periods (tens of injections). These drifts in both retention time and mass accuracy are detrimental for platforms such as LC-MS that depend on these parameters for identification [34]. A drift in retention time can occur when a large number of metabolite extracts is measured in one run on the LC-MS platform or during different analytical blocks that need to be pooled afterwards. This can result in sets of peaks of a single metabolite being considered as belonging to two different compounds in the peak matching step. Re-alignment of the retention time over different samples with the OBI-Warp tool [63] followed by gap-filling (secondary peak picking step to retrieve missing signals within a specified retention time and mass window from the raw data files) [64] can be applied and will significantly reduce the number of double identifications in the eventual list of identified compounds (Figure 3). To handle drift in mass calibration, ubiquitously detected contaminants of known exact mass can be used for internal mass calibration or to align spectra after the unavoidable mass drift during long term studies [12]. One can also include replicate measurements of a series of authentic standards (e.g. the ones used for the above mentioned QSSR model) covering the whole mass range of interest, which will allow recalibrating during data processing. When large metabolomics studies divided over a series of analytical blocks cannot be avoided, normalization of the data can be considered. Dunn *et al*. suggest using a standard quality control sample representative of the sample type under analysis to allow for signal correction within and between analytical blocks [34]. This kind of normalization is model-driven, where an external model is extrapolated to the dataset of interest. More data-driven normalization methods, which originate from microarray studies and have already been applied on NMR metabolomics data [65], are currently under evaluation on LC-MS metabolomics data (unpublished data).

## 6. Outlook and Concluding Remarks

Metabolomics has been a new rising star during the last few years, but as is the case for many new innovative platforms, there naturally is still a lot of room for optimization. For instance, the LC-MS platform could further benefit from technical improvements for more stable column design (to minimize inter-column variation) while awaiting the next-generation mass spectrometers with even higher resolving power, mass accuracy and multiple fragmentation techniques. Although the data processing step is becoming more accessible to non-specialists, efforts to develop (semi-) automated tools for data processing and user-friendly interfaces (e.g. IDEOM) are still highly needed. Last but not least, the increase of large-scale metabolomics projects urges the further development of normalization methods to pool results of different samples that need to be compared.

It is clear that studying the metabolome, lying closest to the phenotype, together with other global molecular profiles, such as the genome, transcriptome or proteome, can significantly enhance our insights into the interactions between the different components of biological systems and how these interactions give rise to a specific behavior of that system and result in a phenotype [66]. Furthermore, the integration of metabolomics and genomics datasets will also contribute to differentiate ‘driver’ mutations from biologically neutral ‘passenger’ changes. However, the integration of different molecular profiling datasets into one comprehensive, easily consultable entity requires an even greater deal of bioinformatics. Even in arcane areas of biology, such as the research on protozoan parasites causing neglected tropical diseases, a rich tradition of metabolomics research has accumulated in a surprisingly short time [12, 13, 24, 25, 28–31, 33, 36, 37, 39, 40, 65, 67]. This minireview, illustrating the application of LC-MS metabolomics with examples selected from *Leishmania* parasite studies, from sampling to data-analysis, highlighted the advantages of the LC-MS platform, but also possible pitfalls which can affect metabolomics research on other (complex) biological systems as well. Untargeted metabolomics studies have shown to disclose complete pathways that respond to drug action, contributing to unraveling the mode of action of these drugs [36]. We expect that even deeper insights into more complex phenotypes, especially the vexing issue of emerging drug resistance, will be provided by carefully designed metabolomics studies in the coming years.
